# Changes of Anaerobic Power and Lactate Concentration following Intense Glycolytic Efforts in Elite and Sub-Elite 400-meter Sprinters

**DOI:** 10.5114/jhk/186074

**Published:** 2024-04-15

**Authors:** Andrzej Mastalerz, Monika Johne, Anna Mróz, Aleksandra Bojarczuk, Petr Stastny, Miroslav Petr, Dominik Kolinger, Anna Pisz, Pavlina Vostatkova, Ewelina Maculewicz

**Affiliations:** 1 Faculty of Physical Education, Jozef Pilsudski University of Physical Education in Warsaw, Warsaw, Poland.; 2 Faculty of Physical Education, Gdansk University of Physical Education and Sport, Gdansk, Poland.; 3 Faculty of Physical Education and Sport, Charles University, Prague, Czech Republic.; 4 Department of Laboratory Diagnostics, Military Institute of Aviation Medicine, Warsaw, Poland.

**Keywords:** fatigue, competition, blood lactate, sprinting

## Abstract

400-m races are based on anaerobic energy metabolism, they induce significant muscle fatigue, muscle fiber damage, and high blood lactate (LA) concentration. Despite extensive research on sprint training, our understanding of the training process that leads to world-class sprint performance is rather limited. This study aimed to determine differences in LA concentration and anaerobic power using jumping tests after an intense glycolytic effort in a group of elite and sub-elite 400-m runners. One hundred thirty male runners were divided into two groups: elite (n = 66, body mass = 73.4 ± 7.8 kg, body height = 182.1 ± 6.2 cm, age = 20.8 ± 4.0 y) running the 400-m dash below 50 s and sub-elite (n = 64, body mass = 72.0 ± 7.1 kg, body height = 182.1 ± 5.2 cm, age = 20.8 ± 4.0 y) with a 400-m personal best above 50 s. The power of the countermovement and the sequential squat jumps was measured in two sets after a warm-up, followed by two intermittent 30-s Wingate tests. LA concentration was measured eight times. It was observed that elite athletes achieved significantly higher power in both types of jumps. The maximum post-exercise LA concentration was significantly lower in the sub-elite group after the 3^rd^, the 6^th^, the 9^th^, and the 20^th^ min after the cessation of two Wingate tests (p < 0.001). The rate of LA accumulation after exercise and the rate of LA utilization did not differ between the groups. It can be concluded that elite and non-elite runners differ in higher LA production but not in LA utilization. Anaerobic power and LA concentration seem to differentiate between 400 elite and sub-elite performance.

## Introduction

The 400-m dash is an athletic event requiring a combination of maximum running speed, which must be achieved and maintained throughout the race (Okudaira et al., 2019). During 400-m sprinting, the anaerobic contributions in men average 63% and represent a critical factor in achieving high performance (Hill, 1999; Nummela and Rusko, 1995). In this event, a runner must reach a very high velocity and preserve the optimal velocity despite intense fatigue. Moreover, it is possible to predict 400 m sprint time based on the anaerobic endurance capacity of athletes (Weyand et al., 1994). For these reasons, understanding the physiological responses to this effort and its relationship with power results during biomechanical testing is critical to performance as it allows to identify areas for improvement to achieve world-class performance.

Sprint performance is significantly influenced by genetic characteristics, with variations among individual athletes being less pronounced than the typical variability observed in external conditions such as wind and monitoring methodologies (Eynon et al., 2013; Maciejewska-Skrendo et al., 2019; Siembida et al., 2021; Yang et al., 2017). Despite these genetic predispositions, key performance determinants such as power, technique, and sprint-specific endurance are trainable aspects (Haugen et al., 2019). Sustaining a high relative velocity for as long as possible is essential for success (Jones and Whipp, 2002), but it is assumed that completing the entire 400-m race at maximum effort is not possible (Hanon and Thomas, 2011; Willis et al., 2012). Some recommend a high-velocity start (van Schenau et al., 1994), while others recommend a more contained and progressive first 200 m to delay rapid lactate production and muscle acidosis (Arcelli et al., 2008). The divergence in recommendations raises questions about the optimal sprinting strategy and its impact on physiological responses, such as lactate production and fatigue. Coaches and athletes try to improve the anaerobic energy system through speed endurance training (Rago et al., 2022). Successful performance in a 400-m run necessitates a swift start followed by a reduction in velocity relative to peak velocity during the last 100 m (Hanon et al., 2011). The fast-start procedure induces higher oxygen uptake and blood lactate concentration (Bishop et al., 2002). This significant anaerobic contribution may influence the decline in velocity during the final 100 m of the race (Hanon and Gajer, 2009). The blood lactate (LA) measurements are used to determine the contribution of anaerobic metabolism, exercise intensity, the lactate threshold, and recovery assessment, enabling a better understanding of the metabolic demands of different activities. The measurement of lactate concentration after exercise provides information about the workload and its impact on recovery (Goodwin et al., 2007).

The need for high LA tolerance is a necessary factor influencing the 400-m dash. However, whether LA production, LA utilization, speed, or power is the differentiating factor between elite and sub-elite 400-m dash athletes has not yet been fully explored. The effect of training adaptation, primarily the ability to generate greater power in the lower limbs, should include the ability to accumulate higher lactate production and buffer H^+^. Therefore, this study aimed to determine differences in lactate concentration in response to maximum anaerobic exercise in a group of 400-m runners, further divided into groups according to their performance. The relationship among maximum power, lactate concentration during exertion and the time of the 400-m run was also investigated.

## Methods

The experimental design employed in this study was a cross-sectional case-control approach aimed at investigating the relationship between athletes’ power and their immediate response to a high-intensity glycolytic load with particular emphasis on post-exercise lactate production and clearance. Health questionnaires were administered before the body mass and height measurement and a standardized warm-up. Subsequently, assessments included countermovement jumps (CMJs), sequential squat jumps (SSJs), and a loading protocol including two repeated all-out anaerobic Wingate tests on a bicycle ergometer. Athletes underwent a 5–6-min warm-up at 1 W/kg and a cadence of 60 revolutions per minute (rpm), with two accelerations of 3–5 s, before conducting the jump and Wingate tests.

### Participants

The study involved 130 male elite athletes from Poland and the Czech Republic. The exclusion criteria were intense training in the preceding 72 h, injury in the past 3 months, and lack of approval from a sports doctor. The inclusion criteria for the experimental group were running a distance of 400 m below 50 s for the elite group and above 50 s in the sub-elite group in the current season, five workouts per week, and participating in 80% of club training sessions. Those who were eligible were included in the elite group (n = 66, body mass = 73.4 ± 7.8 kg, body height = 182.1 ± 6.2 cm, age = 20.8 ± 4.0 y) with 25 athletes from Poland and 41 from the Czech Republic, and the sub-elite group (n = 64, body mass = 72.0 ± 7.1 kg, body height = 182.1 ± 5.2 cm, age = 20.8 ± 4.0 y) with 23 athletes from Poland and 41 from the Czech Republic. In the season in which the research was performed, the average time of the 400-m race for all runners was 49 ± 1.4 s, and it was in the range of 46 to 49 s in the elite and 50 to 52 s in the sub-elite group. Before the study began, all participants were informed about the purpose of the study, the procedures to be applied, as well as potential risks and benefits. All participants completed a survey questionnaire.

### Measures

During both CMJs and SSJs, participants kept their hands on their hips to remove the influence of the arm movement. Participants performed two sets of CMJs and SSJs at their chosen foot position. SSJs were performed in sequences of four squat jumps to a 90° knee joint angle, measured by goniometry. The starting position was marked with a wire behind the participant’s thighs so that participants were aware of the lowest position before jumping upwards. They were instructed to jump dynamically to reach their maximum height. Ground reaction forces were collected using a 600 × 400 mm force plate (Kistler Group, Switzerland, model 9281EA). Force data were collected at 500 Hz. Ground reaction data were used to identify the power of the CMJ and the SSJ during the driving phase using methods recommended previously (Chavda et al., 2018; McMahon et al., 2018). After the evaluations were completed, the raw force plate data were analyzed using Microsoft Excel (Chavda et al., 2018). LA concentration was measured 8 times: at rest, one minute after the warm-up, in the third minute of rest after the first Wingate test, immediately after the second Wingate test, and at the 3^rd^, the 6^th^, the 9^th^, and the 20^th^ min into recovery. Blood was taken from the fingertip. The samples were collected into sodium heparin-treated end-to-end capillaries. LA concentration was assessed with the Biosen apparatus, which uses chip sensor technology and measures LA concentration within the 0.5–40 mmol/l (5–360 mg/dl) range (Biosen C-Line Lactate analyzer, EKF Diagnostics). The following variables of LA kinetics were calculated based on individual LA concentration from the 1^st^ to the 20^th^ min: the peak of LA concentration (LA_max_), the ratio of LA accumulation from the 1^st^ to the 3^rd^ min (ΔLA_1'–3'_), the ratio of LA concentration from the 3^rd^ to the 6^th^ min (ΔLA_3'–6'_) and from the 6^th^ to the 9^th^ min (ΔLA_6'–9'_) and the ratio of LA clearance from the 9^th^ to the 20^th^ min (ΔLA_9'–20'_). The ratio of LA accumulation was obtained as the slope of linear regression in the proper time range. The power of the Wingate test was measured during the loading protocol for the maximal glycolytic response by two repeated all-out anaerobic Wingate tests on a bicycle ergometer (Monark 894 E peak bike, Sweden). It was crucial to attain the highest possible power output to achieve the most effective results on the muscular system and its response to glycolytic exercise. To this end, maximum power values were used in all measurements for further comparisons between groups and to evaluate any potential correlations.

### Wingate Loading Protocol

After the jump tests, participants rested for 5 min. The Wingate test was chosen due to prior research supporting its effectiveness in eliciting maximal engagement of anaerobic metabolism. Previous research has shown that a single 30-s Wingate sprint can reduce muscle glycogen stores in the vastus lateralis muscle by 20–30% (Metcalfe et al., 2012). The applied resistance was set at 7.5% of athletes’ body mass; participants remained seated during the test duration and started from a complete standstill position. Once the load (7.5% of body mass) was preset, the participant’s feet were fixed to the pedals. They were then given a 5-s countdown and began the test with maximal effort. Throughout testing, power was monitored. Participants pedaled as fast as possible for 30 s. Afterwards, they had a 4-min passive rest interval (Lopez et al., 2014) and then performed a second test following the same procedure. After completing the second Wingate test, participants were instructed to take an active rest on a cycling ergometer for 20 min and then LA concentration was determined.

Loading protocols were completed in the preseason period. All testing was carried out in the Physiological Laboratory of the Department of Biomedical Sciences at the University of Physical Education in Warsaw and in the Biomedical Laboratory at the Faculty of Physical Education and Sport at the Charles University.

The study protocol along with the informed consent form were approved by the Bioethics Commission of the District Medical Chamber in Gdansk (approval code: KB-2/21; approval date: 3 February 2021). All the procedures were carried out in accordance with the Declaration of Helsinki developed by the World Medical Association (2013).

### Statistical Analysis

All data were examined to ensure they met the requirements of normality and homogeneity of variance using the Kolmogorov-Smirnov and Shapiro-Wilk tests. To examine the intra- and inter-group differences, analysis of variance (ANOVA) was used. The Fisher's Least Significant Difference

(LSD) test was applied post hoc to make adjustments. All calculations were performed using Statistica (version 13.1) and the level of significance was set at *p* < 0.05.

## Results

Maximal power (P_max_) of the Wingate (W) and jump (CMJ and SSJ) tests was significantly higher in the elite group (Table 1). P_max_ in the second Wingate test (W2) in both groups was lower (*p* < 0.001) and non-significantly different (*p* = 0.140).

**Table 1:** Mean (± SD) variables of the Wingate and jump tests in elite and sub-elite groups.

	Group		ANOVA effects		
	Elite	Sub-elite	Group	Time	Group x Time
P_max_ (W1) [W/kg]	14.7 (1.7)	13.5 (2.1)	F(1, 128) = 9.26, η^2^ = 0.067	F(1, 128) = 110.62, ***p*** < 0.001, η^2^ = 0.464	F(1, 128) = 4.9, ***p*** = 0.028, η^2^ = 0.038
P_max_ (W2) [W/kg]	12.7 (1.6)	12.3 (1.7)			
P_max_ (CMJ) [W/kg]	38.4 (5.7)	34.1 (5.5)	F(1, 128) = 19.81, ***p*** < 0.001, η^2^ = 0.050		
P_max_ (SSJ) [W/kg]	38.3 (5.6)	34.8 (5.4)	F(1, 128) = 13.328, ***p*** < 0.001, η^2^ = 0.038		

**Figure 1:**
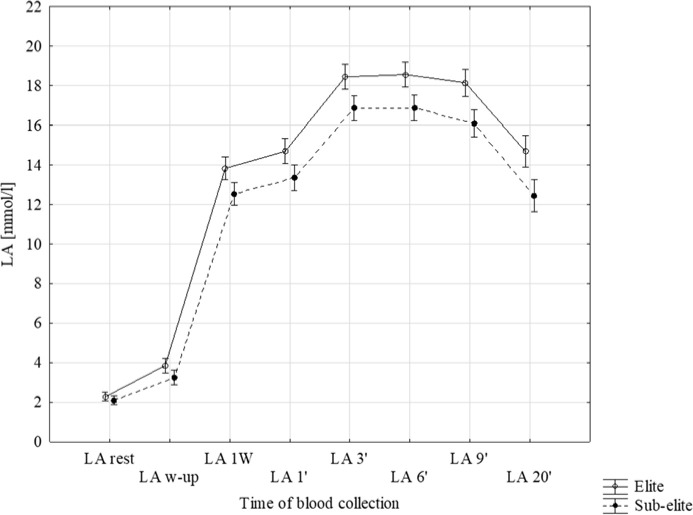
Average LA values in particular minutes of collection. LA rest: at rest, LA w-up: after the warm-up, LA 1W: after the first Wingate test, LA 1' to LA 20': subsequent minutes after the end of the second Wingate test.

Significantly higher LA concentration in subsequent time points was observed in elite sprinters (*p* < 0.001), except for rest and post warm-up time (*p* < 0.05, Figure 1). After the first Wingate test (LA 1'), LA accumulation was approximately 9% higher (*p* = 0.004) up to the 6^th^ min after exercise in the elite group. The kinetics of LA clearance after the 9^th^ and the 12^th^ min were higher by 3 and 5% after the 9^th^ and the 20^th^ min in the sub-elite group. LA concentration at the 20^th^ min was similar to that at the 1^st^ min in both groups (elite *p* = 0.974 and sub-elite *p* = 0.807).

In 40% of participants from the elite and sub-elite groups, the peak lactate concentration (LA_max_) was obtained 3 min after the second Wingate test. The remaining 32% from the elite and 38% from sub-elite groups achieved LA_max_ in the 6^th^ min. However, the time of LA_max_ onset was not found to differentiate between both groups (F(2, 124) = 0.714, *p* = 0.492). The kinetics of LA concentration were assessed in three intervals (Figure 1). The kinetics of LA accumulation after the second Wingate tests (ΔLA_1'–3'_) and clearance from the 9^th^ to the 20^th^ min (ΔLA_9'–20'_) were similar in both groups (Table 2). Non-significant differences were only observed from the 3^rd^ to the 9^th^ min.

**Table 2:** Mean (± SD) variables of LA kinetics in elite and sub-elite groups.

	Group		ANOVA effect
	elite	sub-elite	Group
LA_max_ [mmol/l]	19.1 (2.4)	17.5 (2.8)	F(1, 128) = 12.069, *p*< 0.001
ΔLA_1'–3'_ [mmol/l/min]	1.2 (0.7)	1.2 (0.7)	F(1, 128) = 0.387, *p*= 0.535
ΔLA_3'–6_' [mmol/l/min]	0.04 (0.30)	0.006 (0.43)	F(1, 128) = 0.279, *p*= 0.597
ΔLA6_'–9'_ [mmol/l/min]	−0.14 (0.41)	−0.26 (0.41)	F(1, 128) = 2.621, *p*= 0.108
ΔLA_9'–20'_ [mmol/l/min]	−0.36 (0.1)	−0.36 (0.1)	F(1, 128) = 0.066, *p*= 0.799

LA_max_ significantly correlated with LA kinetics except for the 3^rd^ to 6^th^ and 6^th^ to 9^th^ min intervals, which did not significantly differentiate between the two groups (Table 3). Significant relationships were also observed between maximal power for all tests except the second Wingate test and LA_max_. We also found a significant correlation among running time (t400) and maximum power for all tests, LA_max,_ and LA kinetics from the 1^st^ to the 3^rd^ min (ΔLA_1'–3'_), except P_max_ of the second Wingate test (P_max_ (W2)) and LA kinetics from the 3^rd^ to the 6^th^, the 6^th^ to the 9^th^, and the 9^th^ to the 20^th^ min (Table 3).

**Table 3:** Pearson correlation coefficients and the level of significance (p) for lactate kinetics, maximal power, and time of the 400-m race for all tested athletes.

	LAmax	ΔLA _1'–3'_	ΔLA _3'–6'_	ΔLA _6'–9'_	ΔLA _9'–20'_	P_max_(W1)	P_max_(W2)	P_max_(CMJ)	P_max_(SSJ)
LA_max_		0.33 *p*= 0.001	0.08 *p*= 0.381	0.02 *p*=0.829	−0.19 *p*= 0.027	0.35 *p*= 0.001	0.20 *p*= 0.024	0.25 *p*= 0.004	0.30 *p*= 0.001
t400	−0.29 *p*< 0.001	−0.18 *p*= 0.040	0.03 *p*= 0.721	−0.12 *p*= 0.187	0.08 *p*= 0.339	−0.20 *p*= 0.022	−0.12 *p*= 0.159	−0.36 *p*= 0.001	−0.32 *p*= 0.001

## Discussion

This study aimed to determine differences in lactate concentration as the effect of a maximum anaerobic exercise load in 400-m runners divided into two groups according to their performance. Moreover, the association among maximum power, lactate concentration, and the 400-m run time was estimated. When exercise is repeated over weeks, numerous adaptations occur within the muscle, such as metabolic pathways of energy, which are associated with the ability to remove the accumulation of metabolites after fatigue-inducing efforts (Hanon et al., 2012). It is interesting to note that there are contradictory findings in the literature about the effects of sprint training on adaptation. Although sprint training has a significant anaerobic component, one study discovered that anaerobic metabolism indices were not enhanced during exhaustive exercise after training, suggesting aerobic adaptations (Harmer et al., 2000). However, this might result from prolonged exercise applied in that study considering that a post-training sprint test occurred after the initial sprint. Elite 400-m sprinters reach LA concentration of approximately 20 mmol/l following a competition. This indicates the predominance of anaerobic metabolism and fast twitch muscle fiber recruitment during a prolonged sprint. Thus, elite athletes have a higher glycolytic capacity and can produce more ATP via anaerobic metabolism, which results in higher post-exercise LA concentration. Lower-level athletes have a limited glycolytic capacity and a lower buffering ability of the blood and muscles (Hirvonen et al., 1992; Ohkuwa and Miyamura, 1984). Other studies suggest that adaptation to sprint training is dependent on the duration of sprinting, recovery between sprint repetitions, total volume, and frequency of training bouts, and these variables have profound effects on the metabolic, structural, and performance adaptations induced by a sprint-training program (Iskra et al., 2021; Ross and Leveritt, 2001). It was also proven that in the 400-m race, differences in race strategy might affect physiological reactions (Hanon and Gajer, 2009). Elite athletes can achieve higher absolute and relative speeds (a percentage of their best 200-m result). In addition, world-class athletes pesent a more significant loss of speed in the second half of the race. From a training point of view, this means that elite runners need to increase the rate of ATP delivery from anaerobic metabolism (Hanon et al., 2012; Hirvonen et al., 1992). Our results confirm the above conclusions, by higher lactate concentration after exhausting anaerobic exercise in the elite group of sprinters and the ability to generate higher maximum power in each test. Thus, we confirmed the research hypothesis. A surprising result concerns the rate of LA accumulation after exercise. It has been observed that the accumulation rate is related to speed and distance (Hirvonen et al., 1992). In our research, the rate of LA accumulation did not differ between the two groups (ΔLA_1'–3'_). Such differences were only observed from the 3^rd^ to the 6^th^ and the 6^th^ to the 9^th^ min. During these time intervals, lactate concentration did not change significantly, yet the rate of LA clearance was different. This shows that the rate of lactate accumulation was similar, however, differences in peak LA concentration influenced the initial rate of LA clearance. This rate until the 9^th^ min was non-significantly lower in the elite group. However, from the 9^th^ min, we did not observe differences in the rate of LA clearance. Similar conclusions can be found in a study on African and Caucasian athletes (Bret et al., 2013). Mean blood lactate concentration was significantly higher in Africans than Caucasians from the 5^th^ min. Furthermore, blood lactate concentration rapidly increased to peak LA between the 2^nd^ and the 10^th^ min. However, the estimated LA clearance time was significantly lower at the beginning of rest, yet higher in Africans from the 5^th^ to the 12^th^ min compared to Caucasians (Bret et al., 2013). Therefore, the similarities within these results concern only the accumulation rate, but they are inconsistent with the rate of LA clearance. During a 400-m run, the intensity of exercise will be much higher than during standardized exercise, and it is known that the greater the intensity of exercise, the lower the ability to transport lactate between muscles and blood (Chatel et al., 2016). Previous research has shown that the ability to transport lactate from muscles to blood was related to performance during high-intensity exercise (Bret et al., 2013). This should explain the differences among the results from tests carried out under different conditions despite using a highly strenuous anaerobic protocol. Additionally, Tomschi et al. (2018) found no difference in the rate of lactate accumulation by red blood cells in trained and untrained people. However, other lactate clearance mechanisms may contribute to differences in the clearance rate, such as improved hepatic gluconeogenesis (Sumida et al., 1993), which alters lactate removal in athletes compared to recreationally active individuals. Thus, training status may contribute to a difference in the rate of LA clearance. The accumulation rate is directly related to speed or maximum power, as indicated by significant correlations between power and the rate of LA accumulation. According to the research conducted by Okudaira et al. (2019), the ability to transport lactate into capillaries during sprint interval training protocols with very short recovery time varies among sprinters based on their performance level. Maemura et al. (2004) indicate that the capillary-to-muscle volume ratio is closely linked to anaerobic performance. Therefore, it can be speculated that capillaries will be well developed in the elite group, allowing for more effective lactate clearance even with short recovery periods (Maemura et al., 2004). It should also be noted that the time taken by all sprinters in our research to run 400 m correlated significantly only with the rate of lactate accumulation, suggesting anaerobic influence, and not with the rate of lactate clearance. It is worth mentioning that there is significant variability among individuals in how quickly they clear lactate, which could be influenced by various factors such as individual physiology, training status, metabolic efficiency, and genetics. Generally, LA concentration is considered an effective determinant of running results (Nummela et al., 1992; Okudaira et al., 2019). Fatigue affects a decrease in power (Nummela et al., 1992), and therefore, an increase in LA concentration can be postulated, and a decrease in speed is a consequence of extreme fatigue. It may also be the result of the choice of running tactics. Based on the literature, the contribution of glycolysis to the 400 m may explain the relationship between blood lactate concentration and less aggressive running strategies (Hanon and Gajer, 2009). Therefore, sub-elite athletes exhibiting slower performance in the 400-m race demonstrated lower lactate concentration post exercise and diminished power in all jump tests. This is likely a consequence of employing a different running strategy, potentially impacting the efficacy of both the Wingate tests and jump assessments. This could result from weaker mental commitment or a diminished capacity to perform under fatigue. Moreover, it is noteworthy that the deceleration in speed observed in the last 50 and 100 m is more pronounced in world-class athletes than in athletes at lower performance levels (Hanon and Gajer, 2009). This means that elite runners must be physiologically and mentally able to adopt a more risky strategy than their less experienced counterparts.

## Conclusions

Elite athletes exhibit greater maximum power and greater LA concentration after the Wingate efforts, likely stemming from their adaptation to the predominance of anaerobic metabolism and fast twitch muscle fiber recruitment during a prolonged sprint and more audacious running strategy compared to their less experienced counterparts in the sub-elite group. Despite these differences, the kinetics of LA accumulation and clearance did not differ between the groups studied. Moreover, the time of 400-m running significantly correlated with the rate of LA accumulation from the 1^st^ to the 3^rd^ min after effort and had no relationship with the rate of LA clearance.
